# *Brucella abortus* RB51 lipopolysaccharide influence as an adjuvant on the therapeutic efficacy of HPV16 L1 and HPV16 E7 DNA vaccines

**DOI:** 10.22038/ijbms.2020.51043.11608

**Published:** 2021-01

**Authors:** Masoumeh Shirmohammadi, Hoorieh Soleimanjahi, Zahra Kianmehr, Hesam Karimi, Susan Kaboudanian Ardestani

**Affiliations:** 1 Department of Virology, Faculty of Medical Sciences, Tarbiat Modares University, Tehran, Iran; 2 Department of Biochemistry, Faculty of Biological Science, North Tehran Branch, Islamic Azad University, Tehran, Iran; 3 Immunology Lab, Institute of Biochemistry and Biophysics, University of Tehran, Tehran, Iran

**Keywords:** Adjuvant, Brucella abortus RB51, Cervical cancer, DNA vaccines, Human papillomavirus, Lipopolysaccharide

## Abstract

**Objective(s):**

Human papillomavirus (HPV) is a primary contributing agent of cervical cancer. Eradication of HPV-related infections requires therapeutic strategies. We used* Brucella abortus *RB51 rough lipopolysaccharide (R-LPS) as an adjuvant along with two HPV16 therapeutic DNA vaccines, pcDNA3-E7 and pcDNA3-L1, for improving DNA vaccine efficacy.

**Materials and Methods:**

For evaluation of the *B. abortus* LPS adjuvant efficacy in combination with DNA vaccines to induce cellular immune responses, C57BL/6 mice were immunized with the DNA vaccines, with or without R-LPS adjuvant. IFN-γ and IL-4 cytokines assay was carried out for assessment of cellular and humoral immune responses.

**Results:**

Findings indicated that vaccination with pcDNA3-E7 or pcDNA3-L1 alone could induce strong cellular immune responses, but stronger antigen-specific T-cell immune responses were shown by co-administration of HPV16 E7 and HPV16 L1 DNA vaccines along with R-LPS adjuvant.

**Conclusion:**

Overall, *B. abortus* R-LPS through enhancement of T-cell immune responses can be considered an efficient vaccine adjuvant in future studies and trials.

## Introduction

Cervical cancer is a preventable disease, but it is one of the main causes of cancer-related deaths among women worldwide (1). Annually, there are about 477000 new cases of cervical cancer and approximately 233000 mortalities, with most deaths occurring in developing countries (2). Viral molecular investigations indicated that persistent infection with oncogenic high-risk human papillomavirus (HPV) is the most important etiologic agent of cervical pre-cancer and cancer (3, 4). HPVs are small (52–55 nm), non-enveloped particles consisting of a circular double-stranded DNA genome and icosahedral capsid containing two structural proteins —L1 (major protein, 55 kDa in size; 80% of total viral protein) and L2 (minor protein, 70 kDa) which are required for virion assembly. The expression of L1, alone or in combination with L2, in different expression systems can produce virus-like particles (VLPs) (5-8). The most common high-risk HPV types, particularly HPV16 and HPV18, are the major causes of cervical cancer (9, 10). Therefore, there is a crucial requirement for the production of effectual preventive and therapeutic HPV vaccines to combat this kind of cancer. HPV L1 capsid protein is highly immunogenic, consisting of conformational epitopes for stimulation of humoral immune responses and enabling out assembly into VLPs (11). Currently, two HPV L1 VLP-based prophylactic vaccines, Gardasil® and Cervarix®, have been commercialized for prevention of cervical cancer produced by Merk and GlaxoSmithKline, respectively (12-14). The quadrivalent vaccine, Gardasil, contains HPV VLPs (types 16, 18, 6, and 11) and aluminum hydroxyphosphate sulfate as an adjuvant. Cervarix (bivalent vaccine) also protects against HPV types 16 and 18 and contains ASO4 as an adjuvant (a mixture of monophosphoryl lipid (MPL) A and aluminum hydroxide) (11, 15). Potentially, these vaccines by stimulating conformational L1 epitopes specific humoral immune responses contribute to protection against HPV (16). Although prophylactic HPV vaccines are a great achievement toward diminishing incidence and death rates of cervical cancer, they are not able to eradicate existing HPV-related infections and HPV-associated malignant lesions (17, 18). Therefore, development of potential therapeutic methods is largely desirable for cervical cancer treatment. DNA vaccines are a suitable approach for immunotherapy of cancer and viral diseases. They lead to the clearing of virus-infected cells and tumor cells through effective generation of antigen-specific cell-mediated immune responses (19-21). Thus, this therapeutic strategy can play an important role in the therapy of cervical cancer. The HPV E7 oncoprotein is capable of binding the retinoblastoma tumor suppressor protein (pRb) and through its inactivation leads to driving the cell cycle to cancer. Besides, E7 oncoprotein is constantly expressed in transformed cells and stimulates cellular immune responses (22-26). Several studies have chosen the E7 protein as an ideal target antigen for designing therapeutic HPV vaccines (27-29). One of the strategies for improvement of DNA vaccine efficacy is using an appropriate adjuvant in DNA vaccine formulation. Adjuvants can potentially contribute to enhancing vaccine immunogenicity and accelerating immune response intensity (30). The application of toll-like receptor (TLR) agonists as an adjuvant in combination with the vaccine can lead to improved vaccine efficacy (31). Lipopolysaccharide (LPS), as a pathogen-associated molecular pattern (PAMP), is the external leaflet of the outer membrane in most gram-negative bacteria, which is essential for their growth and survival. LPS consists of three distinct domains in most bacteria: lipid A (a glycolipid portion), oligosaccharide core, and O-specific polysaccharide (a glycan) (32). LPS is a TLR4 ligand that can bind to it through the lipid A portion, then contribute to activation signal transduction and subsequently biosynthesis of pro-inflammatory cytokines such as TNF-α, IL-1 and IL-6, hydroxyl radicals, nitric oxide, and adhesion molecules. Therefore, LPS can be considered an appropriate immunostimulatory adjuvant in DNA vaccine formulation (15, 33).

 Lipid A portion is responsible for LPS endotoxic properties and can result in sepsis and septic shock (15). Therefore, the utilization of LPS as an adjuvant is limited in vaccine formulation. For this reason, several investigations were undertaken for reducing LPS endotoxicity property without diminishing adjuvant activity. Research has led to production of MPL (3-0-desacyl-4-monophosphoryl lipid A), as a component of Cervarix HPV vaccine adjuvant, which is a less toxic form of *Salmonella* *minnesota* R595 LPS (34-36). An investigation demonstrated that *Brucella abortus* possesses a non-classical LPS that is less toxic and non-pyrogenic in comparison with *Escherichia coli* classical LPS. In this study, *B. abortus* LPS in comparison with *E. coli* LPS was less potent in triggering fever in rabbits, killing mice, and producing IL-1β and TNF-α (37). For these specific properties, *B. abortus* LPS can be considered a part of vaccine formulation. *B.* *abortus* RB51 strain has a mutant LPS without O-specific polysaccharide which is called rough LPS (R-LPS) (15). Here, we investigated the effects of *B. abortus* RB51 R-LPS as an adjuvant in combination with HPV16 E7 and HPV16 L1 DNA vaccines in a tumor mouse model for improvement of DNA vaccine potency.

## Materials and Methods

***Plasmid DNA vaccines and adjuvant***

The plasmid DNA constructs, pcDNA3-E7, and pcDNA3-L1 were generated as previously described (38). pcDNA3s were prepared on a large scale by QIAGEN Plasmid Maxi Kit. Agarose gel electrophoresis was performed for evaluating the purity of extracted plasmid DNA. R-LPS from *B. abortus *RB51 was purified by Moreno *et al.* method as previously described (39-41). The degree of purity and the quality of extracted R-LPS were confirmed by sodium dodecyl sulfate-polyacrylamide gel electrophoresis (SDS-PAGE). LPS-speciﬁc silver staining was done according to Tsai and Frasch (42).

***Tumor cells culture***

TC-1 tumor cells were cultured in RPMI-1640 (Gibco) medium supplemented with 10% fetal bovine serum (FBS), 100 IU/ml penicillin, and 100 μ*g*/ml streptomycin at 37 °C with 5% CO_2_ atmosphere.

***Mouse animal model***

Five to six-week-old female C57BL/6 mice were purchased from the Pasteur Institute of Iran and were kept under proper standard conditions in the laboratory animal facility of Tarbiat Modares University. In the current study, all procedures were conducted according to the ethical principles of Institutional Animal Care and Use Committee of Tarbiat Modares University.

***Animal immunization***

For evaluation of vaccine therapeutic effects, the tumor cell suspension consisting of 10^6 ^TC-1 cells in 100 μl PBS was subcutaneously inoculated in the right flank of each mouse. The mice were divided into nine experimental groups with 5 mice in each group. Ten days after TC-1 cell inoculation and development of very small tumors, based on the program presented in Table 1, different vaccine formulations in a total volume of 100 μl were prepared and injected subcutaneously. Then vaccinated mice received two boosters at two weeks intervals. Periodically, tumor progression was monitored, and the tumor volume was calculated according to Carlsson’s formula by measuring the smallest and largest tumor diameters. Two weeks after the last immunization, the mice were exterminated and their spleens were removed for assessment of immune responses by cytokine assay.

***Measurement of IFN-γ and IL-4 cytokines by ELISA assay***

Two weeks after the last treatment, spleens from vaccinated mice were harvested. The splenocyte suspensions were obtained by gentle homogenization in RPMI (Gibco). Red blood cells (RBCs) were lysed by incubation in RBC lysis buffer (20 mM Tris, 160 mM NH_4_Cl, pH 7.4) for 5 min at room temperature, and the pelted splenocytes were resuspended in RPMI supplemented with 10% FBS, 2 mM L-glutamine, 100 U/ml penicillin and 100 µg/ml streptomycin. The splenocytes at a concentration of 2×10^6^ cells/ml were seeded in 24-well plates and incubated with proper mitogen (the 5 µg/ml concanavalin A (Con A, Sigma C7275) or 10 µg/ml *E. coli* LPS (Sigma, L2630)) and then incubated at 37 °C with 5% CO_2_. After 3 days, culture supernatants were collected for measurement of IFN-γ and IL-4 cytokines by using commercial DuoSet enzyme-linked immunosorbent cytokine assay kits (R&D system, Minneapolis, MN). Values were presented as pg cytokine/ml (mean±SD, n=5).

***Data analysis***

One-way ANOVA was performed to compare significant variance among the cytokine concentrations and *in vivo* tumor growth experiments. The analysis was followed by Turkey’s post-test. *P*-values < 0.05 were considered significant. All data analyses were carried out using the GraphPad Prism 6.01 software package (La Jolla, CA, USA).

## Results

***Purification of B. abortus RB51 LPS adjuvant***

**Figure 1:**
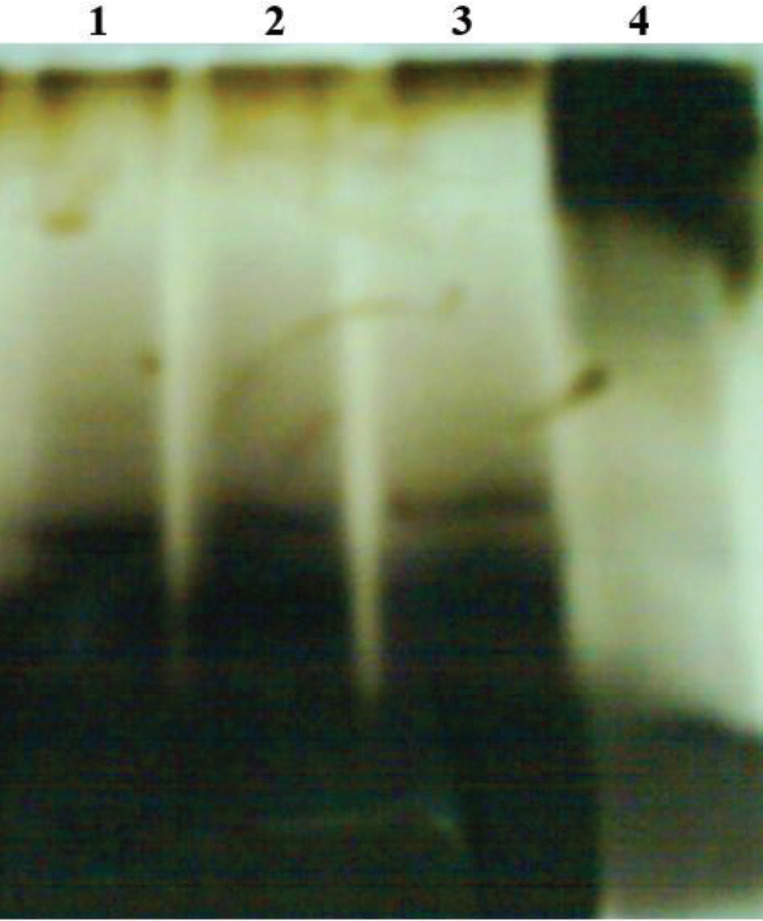
Gel electrophoresis profile of purified R-LPS in special silver staining. Lane 1-3: R-LPS purified from *B. abortus* RB51 and Lane 4: S-LPS purified from *B. abortus* S19 as control. R-LPS due to missing O-side chain polysaccharide had a single diffuse band at the bottom of the gel whereas smooth-LPS purified from *B. abortus* S19 had two distinctive band zones, low and high molecular weight, which were associated with lipids and carbohydrates, respectively

SDS-PAGE patterns of purified R-LPS in special silver-staining was shown in Figure 1. R-LPS had a single diffuse band associated with lipid A in the bottom of the gel, but it did not have a low molecular weight zone associated with carbohydrates portion due to missing O-side chain polysaccharide whereas purified smooth-LPS had two distinctive band zones, low and high molecular weight which was associated with lipids and carbohydrates, respectively.

***Co-injection of R-LPS adjuvant and HPV16-E7 and HPV16-L1 DNA vaccines (ELR group) generate stronger cell-mediated immune responses than other therapeutic groups***

For evaluation of T cell immune responses induced by pcDNA3-E7 and pcDNA3-L1 vaccines and R-LPS adjuvant, tumor-bearing C57BL/6 mice were immunized via subcutaneous injection with any one of the DNA vaccines alone or in combination with R-LPS adjuvant three times with two weeks intervals. Two weeks after the last immunization, spleen cells of vaccinated mice were cultured in the presence of different mitogens *ex vivo* and IFN-γ and IL-4 cytokines secreted by T-cells were assessed using ELISA kits. 

**Figure 2:**
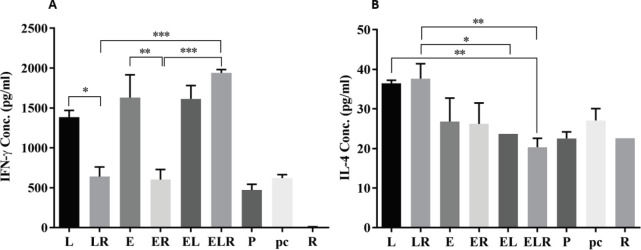
Measurement of cytokine levels secreted by splenocytes of immunized mice. Splenocytes obtained from immunized mice were re-stimulated with appropriate mitogens. IFN-γ and IL-4 levels were determined after induction of cultured splenocytes at 37 °C for three days by commercial ELISA kits. Data are mean±SD, n=5. The levels of statistical significance for differences between experimental groups were determined using ANOVA followed by Turkey’s post-test. Statistical significance was indicated with *P<0.05, **P<0.001, and *** *P*<0.0001. Groups were shown as abbreviations according to Materials and Methods

The results of Th1-cytokine IFN-γ analyses revealed that DNA vaccines alone or together could significantly increase IFN-γ production compared with control groups. As shown in Figure 2A, the DNA vaccines, pcDNA3-E7 or pcDNA3-L1, in combination with R-LPS adjuvant (ER and LR groups, respectively) significantly decreased IFN-γ production compared with mice receiving DNA vaccines without R-LPS adjuvant (E and L groups, respectively) (*P<*0.001). R-LPS adjuvant could not significantly generate IFN-γ response as compared with other groups, and also in combination with each one of the DNA vaccines it contributed to the diminishing of IFN-γ production. Interestingly, mice receiving both two plasmid DNA vaccines and R-LPS adjuvant (ELR group) showed enhanced IFN-γ response compared with EL group mice that received two DNA vaccines without adjuvant, but this enhanced response was not significant. These results represented that cell-mediated immune response (Th1-cytokine IFN-γ) induced by two DNA vaccines can be stronger in combination with the R-LPS adjuvant (ELR group). While this adjuvant in combination with every one of the DNA vaccines results in decreased IFN-γ production. 

In mice receiving pcDNA3-L1 alone or pcDNA3-L1 along with R-LPS adjuvant a significant increase of Th2-cytokine IL-4 was induced compared with other groups. As illustrated in Figure 2B, a combination of R-LPS adjuvant with DNA vaccines did not significantly increase IL-4 production, although there was a slightly increased IL-4 production in the LR group compared with the L group. Also, LR vaccine could induce a significantly increased IL-4 level in comparison with EL and ELR vaccines (*P*<0.001). 

***Co-injection of R-LPS adjuvant and HPV16-E7 and HPV16-L1 DNA vaccines (ELR group) generate potent therapeutic antitumor effects***

**Figure 3:**
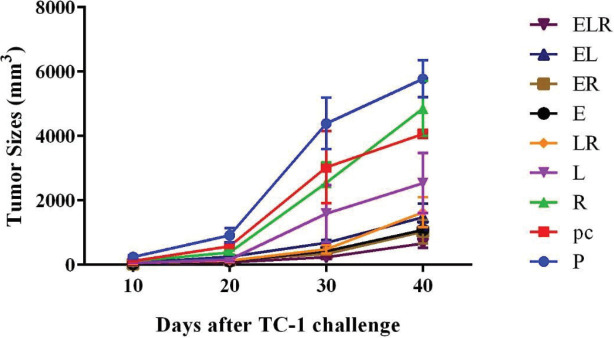
Therapeutic effects of vaccines on the tumor growth rate. For evaluating the therapeutic effects of different vaccine formulations on the tumor growth rate, mice were subcutaneously challenged with 106 TC-1 tumor cells for each mouse. Then tumor-bearing mice were immunized with R-LPS adjuvant and DNA vaccines. Tumor volumes were measured using Carlsson’s formula. Groups were shown as abbreviations according to Materials and Methods

In this study for determining whether R-LPS adjuvant in combination with DNA vaccines could reduce tumor growth rate, C57BL/6 mice were subcutaneously challenged with TC-1 tumor cells in the right flank. Then tumor-bearing mice were treated with DNA vaccines alone or in combination with R-LPS adjuvant three times. As displayed in Figure 3, among the vaccinated groups, the rate of tumor growth in the mice receiving both two DNA vaccines and R-LPS adjuvant (ELR experimental group) was reduced compared with other vaccinated groups. Taken together, these results suggested that using R-LPS adjuvant in combination with HPV16-E7 and HPV16-L1 DNA vaccines is the most effective formulation for the induction of therapeutic antitumor effects of vaccines.

**Table 1:** Experimental groups of mice injected with different vaccine preparations

Abbreviation	Compound (100 μl)	Groups
L	50 μg pcDNA3-L1 alone	**1**
LR	50 μg pcDNA3-L1 along with 10 μg R-LPS	**2**
E	50 μg pcDNA3-E7 alone	**3**
ER	50 μg pcDNA3-E7 along with 10 μg R-LPS	**4**
EL	50 μg pcDNA3-E7 and 50 μg pcDNA3-L1	**5**
ELR	50 μg pcDNA3-E7 and 50 μg pcDNA3-L1 along with 10 μg R-LPS	**6**
pc	50 μg pcDNA3 alone	**7**
R	10 μg R-LPS alone	**8**
P	PBS (Control group)	**9**

## Discussion

The principal criterion of immunization is the generation of proper immune responses for the protection of infection or treatment of disease. In spite of the potent efficacy of available HPV prophylactic vaccines for effectual prevention of HPV-associated infections, they are not capable of clearing existing HPV-related malignant lesions (43, 44). Therefore, an existing therapeutic strategy could be important for eliminating these lesions by enhancing T cell-mediated immune responses. DNA vaccines, as an appropriate therapeutic strategy, have been investigated in several studies that aimed to induce cellular immune responses against HPV-related lesions in the tumor mouse model. In most investigations, E7 and L1 genes of HPV16 are considered as targets for designing therapeutic DNA vaccines (38, 44-46). A study demonstrated that co-injection with HPV16 E7 DNA vaccine and bovine papillomavirus (BPV1) L1 or L2 DNA vector could generate enhanced E7-specific T CD8^+^ cell and potent L1/L2-specific CD4^+ ^T cell immune responses (17). Results of the current study revealed that co-administration of R-LPS adjuvant and two DNA vaccines, pcDNA3-E7 and pcDNA3-L1, could elicit relatively strong cell-mediated immune responses as compared with administration of either pcDNA3-E7, pcDNA3-L1 alone, or pcDNA3-E7 and pcDNA3-L1 together. Besides, tumor growth in mice vaccinated with this vaccine (ELR group) had declined compared with mice vaccinated with two DNA vaccines without the R-LPS adjuvant (EL group). Measurement of cytokine levels demonstrated that IFN-γ production rate was reduced in mice treated with any one of the DNA vaccines, pcDNA3-E7 or pcDNA3-L1, along with R-LPS adjuvant compared with the mice treated with any one of the DNA vaccines alone. Therefore, these results indicated that the use of R-LPS as an adjuvant in co-vaccination with pcDNA3-E7 and pcDNA3-L1 DNA vaccines generated effectively cellular immune responses and treatment antitumor effects stronger than other vaccines formulations. LPS component of gram-negative bacteria is a PAMP and identified as a ligand for TLR4 receptor (47, 48). Recognition of LPS by TLR4 leads to triggering signal transduction and subsequently producing inflammatory cytokines (49). In addition, a previous study reported that the *B. abortus* LPS has much less potential for generation of endotoxic shock (37). Thus, this component of *B. abortus *structure can be considered an efficient candidate for vaccine formulations. Another study indicated the effectiveness of *B. abortus *S19 LPS (S-LPS) as an adjuvant in combination with different PPD fractions from *Mycobacterium tuberculosis* as antigen in skewing of immune responses to Th1 or Th2 pathways depends on the antigen type (50). Studies by Kianmehr *et al.* illustrated that vaccination of mice with S-LPS or R-LPS of *B. abortus* as an adjuvant in combination with HPV16 L1 VLP enhances significantly VLP-specific IgG response compared with mice vaccinated with VLP alone. Furthermore, their study indicated that both S-LPS and R-LPS adjuvants in combination with VLP increased IFN-γ production, and R-LPS in combination with VLP induced stronger IL-4 levels compared with other vaccinated groups (15).

## Conclusion

These findings demonstrate the effectiveness of *B. abortus* LPS as an adjuvant in vaccine formulations consisting of different antigens such as proteins and DNA for improving immune responses. Co-administration of *B. abortus* RB51 R-LPS as adjuvant and HPV16-E7 and HPV16-L1 DNA vaccines lead to generation of strong cell-mediated immune responses and potent therapeutic antitumor effects. Therefore, this research verifies the clinical applications and the prospects of developing HPV16 E7 therapeutic vaccines in combination with immune adjuvants. Overall , we conclude from our results that using R-LPS as an adjuvant in combination with two DNA vaccines (ELR group) improves cellular immune responses and reduces tumor growth rate; this results in enhancement of vaccine therapeutic effects. Therefore, it can be considered an efficient candidate vaccine. In conclusion, our observations may provide valuable prospects for developing a therapeutic approach against cervical cancer through utilization of HPV16 DNA vaccines along with immune-stimulatory adjuvants. 

## References

[1] Lee H-J, Yoon JK, Heo Y, Cho H, Cho Y, Gwon Y (2015). Therapeutic potential of an AcHERV-HPV L1 DNA vaccine. J Microbiol.

[2] Zarchi MK, Heydari E, Tabatabaie A, Moghimi M, Kooti W (2017). Diagnostic value of the care™ HPV test in screening for cervical intraepithelial neoplasia grade 2 or worse. Asian Pac J cancer Prev: APJCP.

[3] Organization WH (2009). Human papillomavirus vaccines: WHO position paper. Wkly Epidemiol Rec= Relevé épidémiologique hebdomadaire.

[4] Shafabakhsh R, Reiter RJ, Mirzaei H, Teymoordash SN, Asemi Z (2019). Melatonin: a new inhibitor agent for cervical cancer treatment. J Cell Physiol.

[5] Bissett SL, Godi A, Beddows S (2016). The DE and FG loops of the HPV major capsid protein contribute to the epitopes of vaccine-induced cross-neutralising antibodies. Sci Rep.

[6] Graham SV (2017). The human papillomavirus replication cycle, and its links to cancer progression: a comprehensive review. Clin Sci.

[7] Senger T, Schädlich L, Gissmann L, Müller M (2009). Enhanced papillomavirus-like particle production in insect cells. Virology.

[8] De Bruijn ML, Greenstone HL, Vermeulen H, Melief CJ, Lowy DR, Schiller JT (1998). L1-specific protection from tumor challenge elicited by HPV16 virus-like particles. Virology.

[9] Sample KM (2020). DNA repair gene expression is associated with differential prognosis between HPV16 and HPV18 positive cervical cancer patients following radiation therapy. Sci Rep.

[10] Zhang T, Xu Y, Qiao L, Wang Y, Wu X, Fan D (2010). Trivalent Human Papillomavirus (HPV) VLP vaccine covering HPV type 58 can elicit high level of humoral immunity but also induce immune interference among component types. Vaccine.

[11] Palmer KE, Jenson AB, Kouokam JC, Lasnik AB, Ghim S-j (2009). Recombinant vaccines for the prevention of human papillomavirus infection and cervical cancer. Exp Mol Pathol.

[12] Wang JW, Roden RB (2013). Virus-like particles for the prevention of human papillomavirus-associated malignancies. Expert Rev Vaccines.

[13] Harper DM, DeMars LR (2017). HPV vaccines–a review of the first decade. Gynecol Oncol.

[14] Einstein MH, Baron M, Levin MJ, Chatterjee A, Edwards RP, Zepp F (2009). Comparison of the immunogenicity and safety of Cervarix™ and Gardasil® human papillomavirus (HPV) cervical cancer vaccines in healthy women aged 18–45 years. Hum Vaccin.

[15] Kianmehr Z, Soleimanjahi H, Ardestani SK, Fotouhi F, Abdoli A (2015). Influence of Brucella abortus lipopolysaccharide as an adjuvant on the immunogenicity of HPV-16 L1VLP vaccine in mice. Med Microbiol Immunol.

[16] Liu DW, Chang JL, Tsao YP, Huang CW, Kuo SW, Chen SL (2005). Co-vaccination with adeno-associated virus vectors encoding human papillomavirus 16 L1 proteins and adenovirus encoding murine GM-CSF can elicit strong and prolonged neutralizing antibody. Int J Cancer.

[17] Yang B, Yang A, Peng S, Pang X, Roden RB, Wu TC (2015). Co-administration with DNA encoding papillomavirus capsid proteins enhances the antitumor effects generated by therapeutic HPV DNA vaccination. Cell Biosci.

[18] Kim TJ, Jin H-T, Hur S-Y, Yang HG, Seo YB, Hong SR (2014). Clearance of persistent HPV infection and cervical lesion by therapeutic DNA vaccine in CIN3 patients. Nat Commun.

[19] Fioretti D, Iurescia S, Rinaldi M (2014). Recent advances in design of immunogenic and effective naked DNA vaccines against cancer. Recent Pat Anticancer Drug Discov.

[20] Donnelly J, Ulmer J (1999). DNA vaccines for viral diseases. Braz J Med Biol Res.

[21] Yang B, Jeang J, Yang A, Wu TC, Hung C-F (2014). DNA vaccine for cancer immunotherapy. Hum Vaccin Immunother.

[22] Dupuy C, Buzoni-Gatel D, Touzé A, Bout D, Coursaget P (1999). Nasal immunization of mice with human papillomavirus type 16 (HPV-16) virus-like particles or with the HPV-16 L1 gene elicits specific cytotoxic T lymphocytes in vaginal draining lymph nodes. J Virology.

[23] Zong J, Peng Q, Wang Q, Zhang T, Fan D, Xu X (2009). Human HSP70 and modified HPV16 E7 fusion DNA vaccine induces enhanced specific CD8+ T cell responses and anti-tumor effects. Oncol Rep.

[24] Lee SY, Kang TH, Knoff J, Huang Z, Soong R-S, Alvarez RD (2013). Intratumoral injection of therapeutic HPV vaccinia vaccine following cisplatin enhances HPV-specific antitumor effects. Cancer Immunol Immunother.

[25] Münger K, Phelps W, Bubb V, Howley P, Schlegel R (1989). The E6 and E7 genes of the human papillomavirus type 16 together are necessary and sufficient for transformation of primary human keratinocytes. J Virology.

[26] Zinckgraf JW, Silbart LK (2003). Modulating gene expression using DNA vaccines with different 3′-UTRs influences antibody titer, seroconversion and cytokine profiles. Vaccine.

[27] Bahrami AA, Ghaemi A, Tabarraei A, Sajadian A, Gorji A, Soleimanjahi H (2014). DNA vaccine encoding HPV-16 E7 with mutation in LYCYE pRb-binding motif induces potent anti-tumor responses in mice. J virol methods.

[28] Demurtas OC, Massa S, Ferrante P, Venuti A, Franconi R, Giuliano G (2013). A Chlamydomonas-derived Human Papillomavirus 16 E7 vaccine induces specific tumor protection. PloS one.

[29] Peng S, Song L, Knoff J, Wang JW, Chang Y-N, Hannaman D (2014). Control of HPV-associated tumors by innovative therapeutic HPV DNA vaccine in the absence of CD4+ T cells. Cell Biosci.

[30] Mohan T, Verma P, Rao DN (2013). Novel adjuvants & delivery vehicles for vaccines development: a road ahead. Indian J Med Res.

[31] Sajadian A, Tabarraei A, Soleimanjahi H, Fotouhi F, Gorji A, Ghaemi A (2014). Comparing the effect of Toll-like receptor agonist adjuvants on the efficiency of a DNA vaccine. Arch Virol.

[32] Molinaro A, Holst O, Di Lorenzo F, Callaghan M, Nurisso A, D’Errico G (2015). Chemistry of lipid A: at the heart of innate immunity. Chem Eur J.

[33] Garcia-Vello P, Speciale I, Chiodo F, Molinaro A, De Castro C (2020). Carbohydrate-based adjuvants. Drug Discov Today Technol.

[34] Thompson BS, Chilton PM, Ward JR, Evans JT, Mitchell TC (2005). The low-toxicity versions of LPS, MPL® adjuvant and RC529, are efficient adjuvants for CD4+ T cells. J Leukoc Biol.

[35] Freytag L, Clements J (2005). Mucosal adjuvants. Vaccine.

[36] Mata-Haro V, Cekic C, Martin M, Chilton PM, Casella CR, Mitchell TC (2007). The vaccine adjuvant monophosphoryl lipid A as a TRIF-biased agonist of TLR4. Science.

[37] Goldstein J, Hoffman T, Frasch C, Lizzio E, Beining P, Hochstein D (1992). Lipopolysaccharide (LPS) from Brucella abortus is less toxic than that from Escherichia coli suggesting the possible use of B abortus or LPS from B abortus as a carrier in vaccines. Infect Immun.

[38] Fazeli M, Soleimanjahi H, Dadashzadeh S (2015). Further stimulation of cellular immune responses through association of HPV-16 E6, E7 and L1 genes in order to produce more effective therapeutic DNA vaccines in cervical cancer model. Iran J Cancer Prev.

[39] Moreno E, Pitt M, Jones L, Schurig G, Berman D (1979). Purification and characterization of smooth and rough lipopolysaccharides from Brucella abortus. J Bacteriol.

[40] Moreno E, Jones L, Berman D (1984). Immunochemical characterization of rough Brucella lipopolysaccharides. Infect Immun.

[41] Kianmehr Z, Ardestani SK, Soleimanjahi H, Fotouhi F, Alamian S, Ahmadian S (2015). Comparison of biological and immunological characterization of Lipopolysaccharides from Brucella abortus RB51 and S19. Jundishapur J Microbiol.

[42] Tsai C-M, Frasch CE (1982). A sensitive silver stain for detecting lipopolysaccharides in polyacrylamide gels. Anal Biochem.

[43] Yang A, Farmer E, Wu TC, Hung C-F (2016). Perspectives for therapeutic HPV vaccine development. J Biomed Sci.

[44] Chabeda A, Yanez RJ, Lamprecht R, Meyers AE, Rybicki EP, Hitzeroth II (2018). Therapeutic vaccines for high-risk HPV-associated diseases. Papillomavirus Res.

[45] Huang Z, Peng S, Knoff J, Lee SY, Yang B, Wu T-C (2015). Combination of proteasome and HDAC inhibitor enhances HPV16 E7-specific CD8+ T cell immune response and antitumor effects in a preclinical cervical cancer model. J Biomed Sci.

[46] Rollman E, Arnheim L, Collier B, Öberg D, Hall H, Klingström J (2004). HPV-16 L1 genes with inactivated negative RNA elements induce potent immune responses. Virology.

[47] Han JE, Wui SR, Kim KS, Cho YJ, Cho WJ, Lee NG (2014). Characterization of the structure and immunostimulatory activity of a vaccine adjuvant, de-O-acylated lipooligosaccharide. PLoS One.

[48] Reed SG, Hsu F-C, Carter D, Orr MT (2016). The science of vaccine adjuvants: advances in TLR4 ligand adjuvants. Curr Opin Immunol.

[49] Campos JH, Soares RP, Ribeiro K, Cronemberger Andrade A, Batista WL, Torrecilhas AC (2015). Extracellular vesicles: role in inflammatory responses and potential uses in vaccination in cancer and infectious diseases. J Immunol Res.

[50] Jamalan M, Ardestani SK, Zeinali M, Mosaveri N, Taheri MM (2011). Effectiveness of Brucella abortus lipopolysaccharide as an adjuvant for tuberculin PPD. Biologicals.

